# Avoiding the tragedies of parasite tolerance in Darwinian beekeeping

**DOI:** 10.1098/rspb.2024.2433

**Published:** 2025-02-05

**Authors:** Nina A. Sokolov, Mike Boots, Lewis J. Bartlett

**Affiliations:** ^1^ Department of Integrative Biology, University of California Berkeley, Berkeley, CA 94720, USA; ^2^ Centre for Ecology and Conservation, University of Exeter, Penryn TR10 9FE, UK; ^3^ Center for the Ecology of Infectious Diseases, Odum School of Ecology, University of Georgia, Athens, GA 30602, USA; ^4^ Department of Entomology, College of Agricultural and Environmental Sciences, University of Georgia, Athens, GA 30602, USA

**Keywords:** parasite, honeybees, resistance, tolerance, varroa, disease

## Abstract

Bee declines have been partly attributed to the impacts of invasive or emerging parasite outbreaks. For western honeybees, *Apis mellifera*, major losses are associated with the virus-vectoring mite, *Varroa destructor*. In response, beekeepers have focused breeding efforts aimed at conferring resistance to this key parasite. One method of many is survival-based beekeeping where colonies that survive despite significant *Varroa* infestations produce subsequent colonies. We argue that this ‘hands-off’ approach will not always lead to *Varroa* resistance evolving but rather tolerance. Tolerance minimizes host fitness costs of parasitism without reducing parasite abundance, whereas resistance either prevents parasitism outright or keeps parasitism intensity low. With clear epidemiological distinctions, and as honeybee disease dynamics impact other wild bees owing to shared pathogens, we discuss why tolerance outcomes in honeybee breeding have important implications for wider pollinator health. Crucially, we argue that unintentional selection for tolerance will not only lead to more spillover from honeybees but may also select for pathogens that are more virulent in wild bees leading to ‘tragedies of tolerance’. These tragedies can be avoided through successful breeding regimes that specifically select for low *Varroa*. We emphasize how insights from evolutionary ecology can be applied in ecologically responsible honeybee management.

## 1. Introduction

Bees, both managed and wild, have significant agro-economic and cultural value [1,2]. However, managed bees in North America face high annual losses and wild bees have experienced substantial declines in parallel [3]. Losses are owing to a range of factors including habitat loss, pesticide exposure and parasite stressors [3,4]. These stressors compound via biological interactions whereby one stressor leads to heightened vulnerabilities to another. For example, bees exposed to pesticides may be immunosuppressed thereby increasing their vulnerability to parasites and pathogens [5–7]. Bees experiencing infection have higher energetic demands, putting further strain on already limited foraging resources [8]. Solving this multifaceted problem requires action at multiple levels, including efforts to reduce parasite and pathogen burdens.

The role of parasites in managed honeybee (*Apis mellifera*) declines is widely documented, including recent re-emerging viral epidemics [9] interacting with contemporary host-jump events by parasites [10]. Emphasis has been placed on the impact and putative vectoring of certain viruses by the invasive ectoparasitic mite, *Varroa destructor* [11]. These mites are the principal driver of high honeybee overwintering mortality in the USA following their unintended introduction in the 1980s [9,12]. *Varroa* are spread through intraspecific vertical transmission when colonies swarm to establish new colonies, and through horizontal transmission—either directly through drift of individuals between colonies, hive robbing and beekeeping management or indirectly through interactions on flowers [13]. Simultaneously, efforts have highlighted parallel stressors in wild bees in the form of novel or outbreaking parasites [14]. While the interacting effects of poor nutrition and pesticide exposure are partly responsible for observations of declines, newly emerged parasites certainly play a part in underpinning wild bee health concerns ([15]; box 1).

Box 1: ***Varroa*, viruses and honeybees.**

*Varroa* mites are an ectoparasite of *Apis* spp. A host species jump occurred from the Asian honeybee (*Apis cerana*) to the western honeybee (*Apis mellifera*), following which *Varroa* have spread nearly worldwide [9]. *Apis cerana* and *Varroa jacobsoni* have co-evolved for millennia, with *A. cerana* developing complex behavioural adaptations against this parasite to minimize host pathology [16,17]. *Apis mellifera* only began to be parasitized by *Varroa* in the mid−twentieth century and had very limited established defences. Consequently, *Varroa* has caused widespread losses of *A. mellifera* colonies in many parts of the world [18], through their apparent vectoring of viruses [15]. Viral transmission putatively occurs during the parasite’s reproductive stage whilst feeding on developing honeybee pupae [19]. Once pupae emerge as adults, they experience highly virulent viral infections. An example is the suite of physical deformities caused by the vectored deformed wing virus (DWV; [19]). While the parasitic vector can be controlled through beekeeper management and chemical pesticides [20,21], unmanaged or poorly controlled *Varroa* infestations are associated with high virus titres, loss of colony productivity and elevated colony mortality [22,23].

Pathogen spillover occurs when a reservoir host population transmits a disease to a novel host population in a shared environment [24]. Emergent pathogen stressors affecting wild bees probably result from either perpetual dead-end spillover events or recent cross-species transmissions originating from imported managed bees or managed bees that have acquired new parasites [25,26]. Particularly in North America, this is a serious problem with migratory beekeeping for crop pollination leading to the mass transplantation of managed, non-native honeybees from geographically distant locations [27,28]. Novel honeybee-associated pathogens are encountered by wild bees while out foraging [29,30] or have been acquired recently before subsequent intraspecific transmission [31] and exacerbated in severity by pesticide and nutritional stressors [32]. Evidence points towards the proximate role of infected managed honeybees and *Varroa* parasitism in the spillover of these viruses [26,33]. Honeybee health and wild bee health are interconnected in two ways: indirectly through shared stressors and directly through the spillover of honeybee pathogens into wild bee populations. Consequently, the ecological and evolutionary context of pathogen spillover must be included in responsible discussions of honeybee health and bee conservation.

Finding or breeding ‘better bees’ with higher survivorship has been the goal of researchers and practitioners to reduce unsustainable honeybee losses to *Varroa*. As it stands, beekeepers control *Varroa* through miticidal chemical applications [20,21]. It is, therefore, intuitive to breed bees that control *Varroa* and maintain low mite numbers via adaptations such as hygienic behaviours rather than reliance on chemical treatments [34,35]. Consequently, there is widespread academic and applied interest in ‘*Varroa*-resistant’ honeybee stock ([23,36–38]; box 1). This is a worthy goal, often paired with concern over *Varroa* evolving resistance to existing miticidal pesticides [20,39,40]. Only recently has bee-breeding research been framed in the wider context of evolutionary ecology in host–parasite systems [41]. Prominent early work in *Varroa*-surviving bees did differentiate resistance versus tolerance [42], in line with discussions originating in the plant-herbivore literature at the time [43]. However, this distinction has become blurred or lost in recent decades. As different strategies to improve honeybee health in the long term are embraced by practitioners and breeders at large, we must frame these exercises in directed evolution within broader paradigms of host–parasite evolution.

Differentiating outcomes around tolerance, resistance and specific parasites is therefore a necessary next step in bee health efforts worldwide. Box 2 describes how tolerance is understood as a strategy to reduce the host’s cost of parasitism by mitigating the pathology of infection without reducing parasite load. It is fundamentally different from other forms of resistance-based disease defences which rely on avoidance, control or recovery. There is now a rich body of theoretical evolutionary ecology underpinning our understanding of tolerance strategies, including how they arise and their consequences (see box 2). In this review, we discuss how the impact on wild bees through spillover of honeybee viruses will differ based on the selection approach used by beekeepers. Survival can arise from either tolerating high-mite levels—probably through tolerating viral infections—or resistance to mites by reducing their survival or fecundity, leading to smaller mite populations and lower viral loads. ‘Darwinian’ beekeeping approaches were recently popularized amongst beekeepers-at-large by Dr Tom Seeley [48,49]. Seeley’s Darwinian beekeeping method sets a *Varroa* threshold for a colony, with colonies above that threshold culled or otherwise prevented from breeding. This is different from but often confused with survivor-stock selection [50,51] which passively selects for honeybees that survive without thought to parasite populations. Survivor-stock has often been described in the popular beekeeping zeitgeist as a ‘Darwinian’ approach. Tracking parasite populations is critical while breeding honeybees because the mechanistic differences between tolerance and resistance to parasites have significant ecological consequences. Although both mechanisms lead to honeybee survival, breeding methods that neglect parasite monitoring risk evolving highly tolerant hosts and subsequent ‘tragedies of tolerance’, potentially devastating wild bees. Preventing this outcome is paramount for broader pollinator health.

Box 2: **clarifying the conceptual basis of the evolution of defence to pathogens.**

Broadly, host defence can be divided into one of three categories: (i) avoidance, where hosts minimize their chance of either encountering or being infected by a parasite (infections are reduced); (ii) control/clearance, where hosts limit parasite population growth to reduce the cost of being infected (infections are less severe/shortened); and (iii) tolerance, where hosts mitigate the costs of being parasitized without altering the infection course (infections may be common and parasite loads high, but fitness costs small to hosts). Of these, control/clearance are often mechanistically linked and are conceptually very similar. Therefore, avoidance/control/clearance may be grouped as ‘resistance’ since they all reduce pathogen fitness, whereas tolerance does not. Notably, avoidance can include behavioural adaptations that reduce or remove the likelihood of encountering a parasite. This extends our understanding of resistance beyond just immunology/physiology [44], to include preventing infection by avoiding vectors of pathogens.

Tolerance is the most distinct of these from an evolutionary and ecological perspective as it is the only host strategy that reduces vulnerability without imposing a fitness cost on the parasite [45,46]. Moreover, the evolution of tolerance is self-reinforcing if it increases the infectiousness of the host. As tolerance strategies become more common in a population, parasites become more prevalent. The force of infection (‘parasite pressure’) therefore increases, which strengthens the selection pressure on non-tolerant hosts to evolve further increased tolerance to the parasite. This is in stark contrast to the other classes of resistance introduced above. Costly resistance mechanisms experience negative frequency-dependent selection, as a partly resistant population reduces the force of infection by reducing transmission, thus, lowering the selection pressure on susceptible hosts to defend against the parasite [47]. The fixation of tolerance strategies is therefore much easier than other resistance strategies [45,46]. The difference between tolerance and resistance mechanisms is more profound when coevolution is considered, as parasites experience strong selection to overcome avoidance, clearance or control strategies. Conversely, tolerance of a host does not reduce parasite fitness and so does not participate in ‘arms-race’ coevolutionary dynamics, leading to a more evolutionary stable host–parasite relationship.

Box 3: **three tragedies of tolerance.**

Tolerance has been shown to be easy to evolve to fixation. With tolerant hosts, parasites do not experience fitness loss from this strategy, thus coevolutionary-arms-race dynamics that are often thought of in host–parasite evolution do not occur [89]. However, tolerance is also associated with negative ecological outcomes, which have been dubbed 'tragedies of tolerance' [90]. These ‘tragedies’ described below are all relevant to the ecological outcomes of honeybee breeding efforts concerning spillover:

- **increased infection rates at the population level:** Miller *et al*. [89] demonstrated the original tragedy of tolerance, showing that if host–parasite systems fail to co-evolve towards full commensalism, tolerance strategies may reduce individual case mortality but can ultimately increase absolute parasite-driven mortality across the whole population. Tolerance can therefore represent an evolutionary end-state where more individuals are dying from a parasite than was the case when the population was fully susceptible to the pathogen. This ecological phenomenon can be easily extended to include spillover, whereby under the tolerant evolutionary scenario there are simply more numerous infectious definitive hosts in the environment to cause spillover into sympatric species;
- **increased infectiousness at the individual level**: additional tragedies are apparent from the evolutionary response of the parasite to a tolerant host. Miller *et al.* [89] also detail that in response to host tolerance, parasites may evolve higher replication rates owing to the reduction of virulence costs associated with high replication rates under the widely accepted virulence-transmission trade-off framework. An example of this is seen in virulence modulated through immunopathology, whereby evolving tolerance reduces immune response to the pathogen even at high replication rates [91,92]. Ultimately this leads to tolerant hosts being more infectious; and
- **increased virulence in non-focal hosts:** an additional linked tragedy follows from the above parasite evolutionary phenomenon. Parasite strains with very high replication rates are hypervirulent when infecting non-tolerant hosts. It is well demonstrated that higher parasite replication rates are typically associated with greater virulence of infection [93–96] when considered in non-tolerant hosts. Consequently, parasites adapted to a tolerant focal host may replicate at hypervirulent rates when they find themselves infecting non-focal hosts, leading to more severe pathology [89,90].

Taken collectively, these three linked tragedies lead to higher rates of spillover and more severe outcomes of spillover when tolerance of shared parasites is evolved by the definitive host. This is owing to infected definitive hosts being more numerous, more infectious, and infected by pathogen strains which are more virulent when measured in non-tolerant hosts.

## 2. The impact of honeybee viruses on wild bee populations

Interspecific pathogen spillover is important in the context of tolerance where the tolerant species increases the force of infection (transition rate between susceptible and infectious) for non-focal host species. The field of invasion ecology has highlighted the ability of an invasive species to devastate and displace a native competitor via transmission of a pathogen to which it (the invasive) is tolerant but the competitor (the native) is susceptible [52–54]. One of the most studied systems demonstrating this phenomenon is that of invasive, squirrel pox-tolerant grey squirrels (*Sciurus carolinensis*) in the United Kingdom, where they have displaced the native, squirrel pox-susceptible red squirrels (*Sciurus vulgaris*; [55,56]).

Certain honeybee pathogens are nearly ubiquitously found in honeybees [57], exemplified by the high prevalence of deformed wing virus (DWV; [58,59]). Highly abundant pathogens set the stage for the risk of interspecies transmission events via spillover [31,60]. Bee species experience interspecific pathogen spillover through foraging on shared floral resources [29,61–63], especially from abundant, generalist honeybees. This was shown experimentally with DWV successfully being transmitted between honeybees and bumblebees on a shared clover resource [64]. ‘Honeybee’ viruses are found throughout nearly all the major bee families [60,65–68]. However, detection of a virus does not necessarily indicate infection nor the competency of that host. Evidence for replicating viruses in native bees has been found for at least seven ‘honeybee’ viruses [14], but little is known about which species act as primary host reservoirs. Phylogenies show that viruses do not cluster by species and instead cluster by geography, suggesting free dissemination of viruses between species [68,69]. Although not all viruses are associated with *Varroa*, DWV is one of the most common viruses found in honeybees [19], is actively vectored by *Varroa* and is the bee virus found most frequently in other insects thus far investigated [68]. DWV has been experimentally shown to actively replicate in bumblebees infected as larvae via oral inoculation and in adults after injections [70]. Crucially, DWV prevalence in non-*Apis* species is correlated with DWV load and *Varroa* infestation rates in the overlapping honeybee populations [31,71–73]. DWV is of honeybee origin [9], providing evidence that the recent emerging epidemic of the virus amongst non-*Apis* bees has been driven by *Varroa* vectoring the virus in honeybees. Estimating the effect of these pathogens on non-*Apis* bees primarily comes from experimental inoculation studies of *Bombus* that have demonstrated increased host mortality rate [29,74,75].

**Figure 1.**
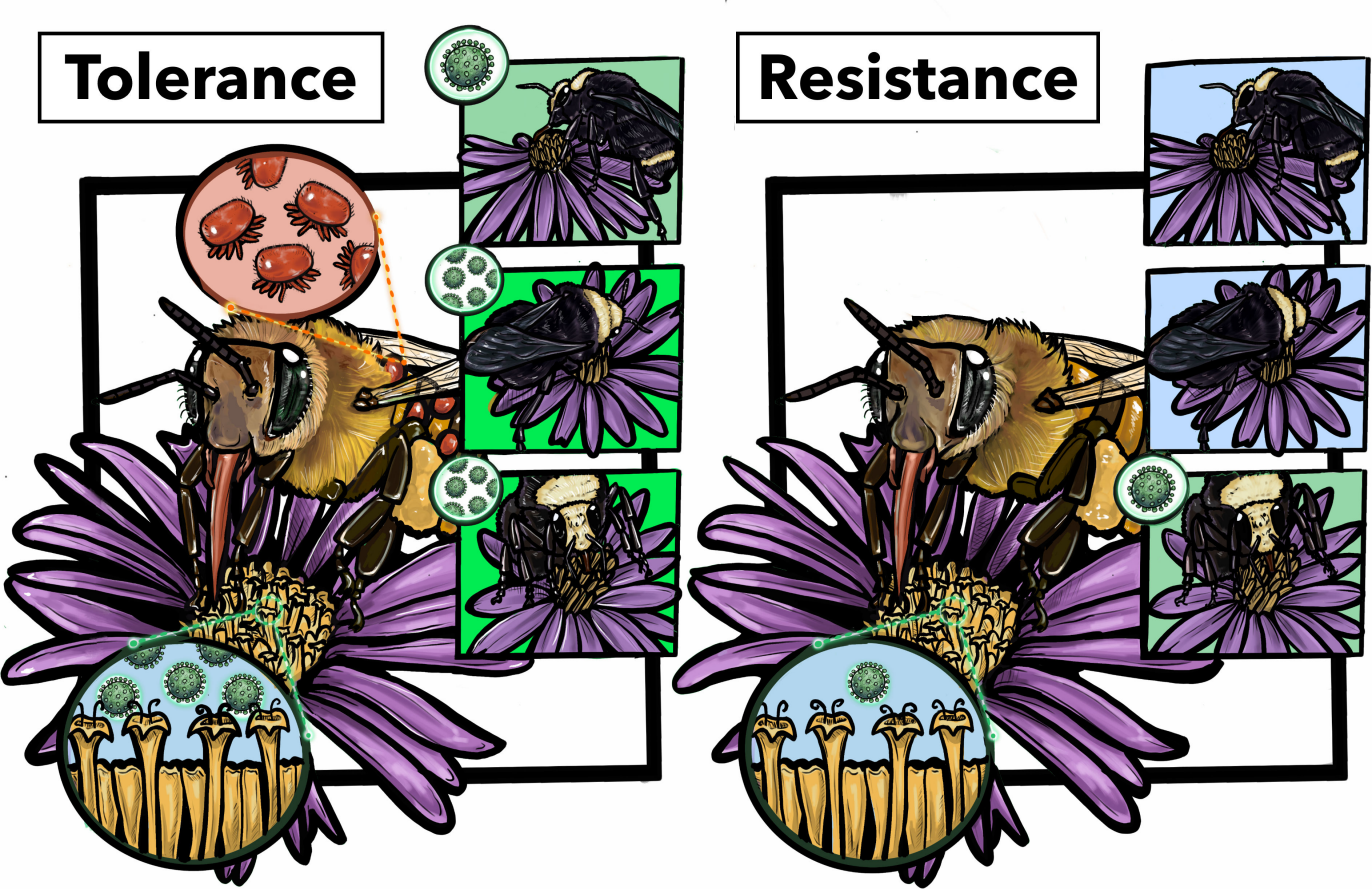
Graphical abstract illustrating the ecological consequences between tolerance and resistance to *Varroa destructor* in honeybees. With parasite tolerance (left) a honeybee shows high *Varroa* (red) infestation, shedding more viruses into the environment (green). This leads to an increased risk of environmental transmission between honeybees and co-foraging bumblebees. In this example, all three bumblebees are infected and two have higher viral loads. Alternatively, (right) a *Varroa*-resistant honeybee is shown free of mites, shedding fewer viral particles into the environment, leading to decreased rates of infection with one infected bumblebee showing a low viral load (green) and two healthy bumblebees (blue).

Although improving honeybee health cannot eliminate the risk of pathogen spillover, reducing the prevalence and severity of infections can significantly reduce infectious propagule pressure on wild bees that coexist with managed honeybees (figure 1). We expect that pathogen load and transmission success are linked as interspecies infections are understood to occur via environmental transmission through foraging on contaminated floral resources [64,66,76]. Illustratively Manley *et al*. [26], compared viral load and prevalence in honeybees and bumblebees on islands in the United Kingdom that differed in their presence of *Varroa*. They found that *Varroa* presence led to higher viral loads and viral prevalence in honeybees as well as in sympatric *Bombus* populations when compared with islands that did not have Varroa. Similarly, Alger *et al*. [25,77] also looked at the spillover of viruses between honeybees and bumblebees and found that the highest viral prevalence in bumblebees was associated with higher viral loads in honeybees.

## 3. Tolerance versus resistance as distinct host defence mechanisms

The study of resistance and tolerance evolution has a long history in plant sciences, where much of the rigorous paradigm was conceived [78–82]. More recently, the integration of tolerance evolution, ecology and immunology has been explored in animals [83–85] alongside resistance. The mechanisms underpinning tolerance and resistance evolution are not well understood, but advances have come with respect to the integration with immunology [86,87], including in insects [88]. Through synthesizing these studies and the body of theory outlined in boxes 2 and 3, we can make important predictions on how resistance and tolerance are likely to manifest in an applied system and the subsequent ecological and evolutionary consequences. Here, we focus on tragedies of tolerance: ecological and evolutionary phenomena associated with tolerance (but not resistance) which in applied contexts are broadly negative.

As in box 3, the original ‘tragedy of tolerance’ was the observation that while individual infection-mortality rates will substantially decline as a host population evolves tolerance, the higher infection rates associated with tolerant populations may mean that the total number of deaths from infections across a tolerant population is higher than in a susceptible population [89,90]. Managed honeybees live at such high densities as to achieve a high prevalence of infection within hives [57], and therefore this original single-population tragedy of tolerance has clear implications for spillover in the honeybee virus system; whereby the force of infection experienced by non-*Apis* wild bees increases as tolerance in honeybees evolves. In addition, the evolution of pathogens in their tolerant hosts is expected to lead to higher virulence when infecting a different species [92]. These predictions are well established in the theory of tolerance evolution [89,91] and have been investigated in specific cases in applied systems such as malaria [97,98] and more recently in bat viruses [99–101]. Pathogen tolerance in bats is enabled by specific immuno-physiological adaptations, leading to elevated virulence of those pathogens when they spillover into new non-chiropteran hosts. We explain the theoretical basis for these tragedies in box 3 verbally and posit that the honeybee–wild bee virus spillover system is an ideal and important test case for these predictions about the nature of tolerance in underpinning virulent cases of disease spillover.

## 4. Tolerance and resistance to viruses and mites in honeybees

**Figure 2.**
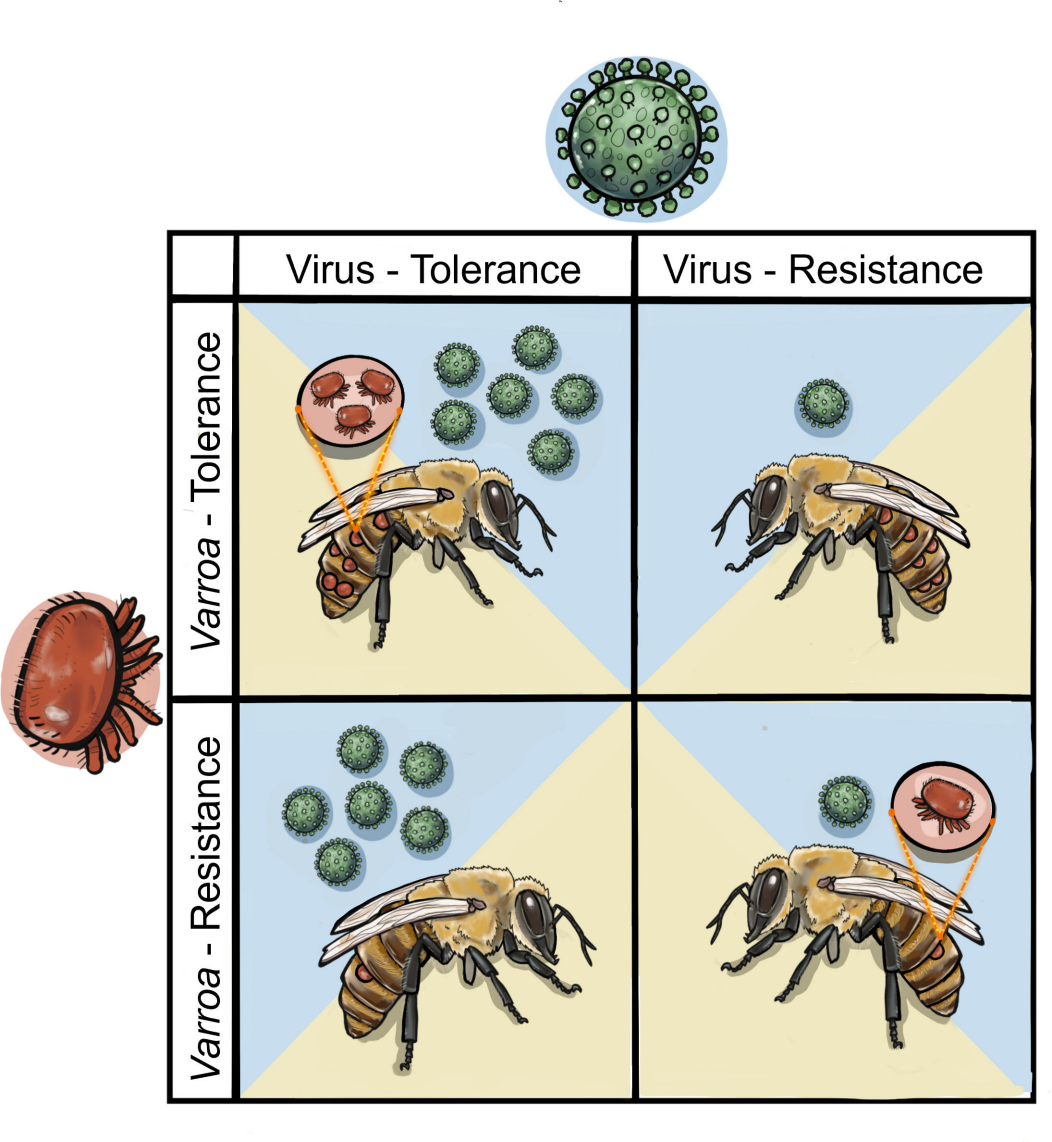
The phenotypic combinations possible in the honeybee hosts resistance/tolerance to *Varroa* (red) and viruses (green). *Varroa*/virus tolerance (top left) is a likely combination of natural selection via undirected ‘survival-stock’ breeding wherein the host tolerates viral infection which by proxy tolerates the mite. The *Varroa* tolerance, virus resistance (top right) and *Varroa* resistance, virus tolerance (bottom left) phenotypes are less supported in the literature. *Varroa*/virus resistance (bottom right) occurs through control of the mite population that controls viral infections by limiting host–vector interactions.

A key consideration of honeybee breeding efforts is the tripartite interaction between the host (honeybee) parasitic vector (*Varroa*) and viral pathogen(s) which underpin major honeybee losses (box 1). While well studied, this system has rarely been examined explicitly using the evolutionary ecology framework of resistance and tolerance as described above. This is illustrated by work such as Thaduri *et al*. [102] who describe a ‘mite-resistant’ honeybee stock from Gotland, but as part of their work identified that the honeybee population was actually tolerant of *Varroa* and their associated viruses. Evidence consistent with tolerance of viruses has been reported from feral honeybee populations in the United States [103,104]. An insightful study published by Penn *et al*. [105] differentiated tolerance, resistance or susceptibility to DWV among honeybee lineages, some of which were bred for resistance to *Varroa*. In figure 2, we outline honeybee breeding for defence against *Varroa* at the intersection between tolerance and resistance split across *Varroa* and viruses. In this simplified example, honeybees are assumed to start susceptible to both and then are selected to be either resistant, tolerant or remain susceptible to viruses, and resistant or tolerant of *Varroa* (figure 2). This is an extension of earlier successful applications of this framework [38,105,106], which we derive from evolutionary ecology principles to avoid the pitfalls of ‘black box’ survival-based selection [50]. We describe these four phenotype combinations below and then discuss core examples in greater detail.

### (a) Virus resistant, *Varroa* tolerant

There is circumstantial evidence from the Brazilian island Fernando de Noronha that honeybees successfully tolerate high mite infestation rates owing to an apparent lack of replicating vectored virus in the population [107]. A similar example has been documented in Papua New Guinea, where the absence of DWV led to a new low-virulence *Varroa* spillover into *A. mellifera* [108]. These examples illustrate the fundamental concept of how an absence of significant viral replication (through avoidance, clearance or control of viruses) allows for the tolerance of *Varroa*, as much of the virulence associated with *Varroa* is through its interaction with viruses [21,109]. However, the evolutionary stability of this state is doubtful. *Varroa*-associated viruses are rapidly evolving and re-emerging pathogens [9,110], and we would predict from evolutionary theory a strong selective pressure on them to overcome resistance mechanisms in the honeybee host, should adequate variants in the virus population arise. The putative immunosuppression of honeybees by *Varroa* also makes this outcome difficult to mechanistically justify [111]. In both cases of these ‘natural experiments’, we would expect a DWV variant capable of being vectored by *Varroa* to rapidly invade and cause mortality in the host (an evolutionary unstable system), making this an implausible long-term management solution.

### (b) Virus tolerant, *Varroa* tolerant

This is conceptually very similar to circumstance (§4a). Honeybees that experience little virulence from viral infection are tolerant of *Varroa* by extension. Theoretically, this is the predictable outcome of natural selection as we outline in box 2, in part owing to the fundamental evolutionary stability. However, this carries substantial risk for wild bees. Recent work from isolated wild populations of *Varroa-* and virus-parasitized honeybees demonstrates this evolutionary outcome in the ‘wild’ [102]. They show that if their ‘mite-resistant’ Gotland bees are challenged with viral inoculum, they are infected by viruses at the same rate as mite-susceptible bees and show infections with the same viral titres as mite-susceptible bees but do not suffer the same viral infection-associated mortality rates. As such, they do not so much ‘resist’ *Varroa* parasitism as they tolerate *Varroa* infestations by not suffering pathology from prevalent high-titre viral infections. This is a clear demonstration of the evolution of parasite tolerance [112]. Further evidence of viral tolerance has been observed in ‘survivor/Darwinian’ bees in the United States [103,104]. Feral honeybee colonies have exhibited elevated viral titres despite predictions that feral honeybees should be *Varroa*-resistant [113]. Even more recently, Penn *et al*. [105,114]demonstrated this same principle by comparing different stocks of *Varroa-*resistant bees and building on the work of Khongphinitbunjong *et al*. [115]. ‘Pol-line’ and ‘Saskatraz’ bees that had been bred partially for either a ‘low-*Varroa’* phenotype or *Varroa-*sensitive behaviour, and correspondingly exert strong control on *Varroa,* were described as susceptible to DWV infections (see further discussion in §4d). Russian bees (originated initially as a ‘survivor-stock’ line from eastern Russia and since maintained as a breeding programme) showed tolerance to DWV infections, exhibiting the same viral loads but far less severe pathology. Overall, from both theoretical understanding and evidence from wild bees, we expect ‘survivor-stock’ breeding programmes [50,51] to be more permissive to tolerance as an evolutionary outcome.

### (c) Virus tolerant, *Varroa* resistant

This unlikely combination of viral tolerance and *Varroa* resistance is a highly speculative incident inspired by comments made in Mondet *et al*. [116]. They posit that during the invasion of *Varroa* in New Zealand, *Varroa* populations declined because of mortality associated with the extremely high incidence of DWV. Parasitic arthropod vectors of pathogens are known to also be engaged in an evolutionary arms race with the pathogens they vector [89,117], which could limit their populations if they are intolerant of infection. Hypothetically, honeybees tolerant of viruses that are virulent when infecting *Varroa* may be resistant to *Varroa* parasitization as a result. Comparable observations have been made in other systems [118]. While we expect the likelihood of this circumstance to be low, Ryabov *et al*. [119] recently showed that *Varroa* infected with and more competently vectoring DWV have significantly reduced lifespans compared with their counterparts, illustrating this as an interesting system in understanding tripartite host–vector–virus evolution.

### (d) Virus ‘resistant’, *Varroa* resistant

This double-resistant outcome is traditionally what bee breeding efforts claim to be pursuing. Measuring resistance is complex and requires assessing *Varroa* population growth rates by quantifying mite fecundity, phoretic/brood infestation and mortality [106]. We argue this is a necessary endeavour, as controlling vector populations prevents transmission of the pathogen of concern. Through an evolutionary ecology lens, the host limiting or eliminating *Varroa* is a resistance mechanism to the virus as well (‘avoidance’—see box 2). However, this case could easily be (mis)characterized in laboratory studies as ‘*Varroa* resistant, virus susceptible’, as any study that ‘bypasses’ the evolved *Varroa* behavioural control mechanisms of honeybees exhibiting this phenotype combination. For example, infecting honeybees with viruses via *Varroa* application or needle injection would cause a highly pathogenic infection. This underpins the results of Penn *et al*. [105], who emphasized that two of their ‘*Varroa*-resistant’ bee stocks exhibited marked vulnerability to DWV infections and so described those bee stocks as DWV susceptible in the context of controlled infection. In an evolutionary context, achieving mite-resistant stock imparts a type of viral resistance *de facto* via avoidance and therefore holds significant promise rather than risk for wider bee health. Evidence already exists for the success and achievability of this approach, highlighted by the many efforts to successfully breed for ‘low-*Varroa’* phenotype bees.

### (e) Real world phenotypes

In reality, there are no clean separations of resistant and tolerant bees to either viruses or *Varroa*. Most well-studied examples of evolutionary responses to *Varroa* show some combination phenotype of both tolerance and resistance. Illustratively, the recent studies of Thaduri *et al*. [102] and Penn *et al*. [105,114] begin to explore parts of this framework by examining the mechanisms underlying honeybee survival to *Varroa* parasitization. Both highlight the evolution of tolerance to DWV in instances of honeybee adaptation to *Varroa* infestation including both a natural selection case (Gotland honeybees) and a breeding programme (Russian honeybees). Per our understanding of the tragedies of tolerance, this should be of ecological and conservation concern. Nevertheless, both examples also show evidence of various behavioural resistance adaptations, such as ‘recapping’ in the Gotland bees [120] and lower worker brood *Varroa* infestation rates in Russian bees [121]. At the same time, they show mechanisms of tolerance such as heightened drone brood production in Russian bees to segregate *Varroa* away from important worker brood. This was discussed early in the Russian breeding programme. Observed *Varroa* tolerance was directly ascribed as a product of unmanaged natural selection (survivor-stock) as early as the turn of the Millennium [42]. Significant evidence from studying feral honeybee populations has also shown honeybee tolerance of viral infection conferring increased tolerance of *Varroa* infestation (figure 2; [103,104]). This includes the well-studied Arnot Forest population [122], which from its initial characterization was described as ‘persisting with *V. destructor’* yet ‘not inhibiting *V. destructor* population growth’ [123]. We classify this as tolerance. The Gotland natural experiment, evidence from the Arnot Forest honeybee population, and elements of the Russian honeybee breeding programme exemplify what we argue is a ‘tolerance-permissive’ outcome of ‘survivor-stock’ breeding through undirected natural selection. This is in agreement with core theory (box 2) and thereby of plausible serious concern to wild bees [112].

By contrast, Pol-line honeybees were lineages selected specifically for mechanism-first breeding to achieve low *Varroa* populations, by selectively breeding colonies with high expression of *Varroa-*sensitive hygienic behaviours (e.g. Pol-line bees, partly selected for a ‘low-*Varroa*’ phenotype). These bees show low tolerance to viruses and exemplify the ‘resistant-resistant’ phenotype we describe, where low viral titres of *Varroa*-associated viruses were observed owing to resistance via avoidance through the bees’ control of *Varroa* [41]. Should *Varroa* populations overcome resistance mechanisms and grow in size, the Pol-line colonies quickly collapse owing to their lack of (viral) tolerance. Such phenotypes do not fall foul of any tragedies of tolerance, and Pol-line bees are one example of many instances of the possible success of this ‘low-*Varroa*’ approach.

In the case of Seeley’s ‘Darwinian’ beekeeping, this ‘black box’ approach selectively breeds *Varroa* resistance with no specific behavioural or physiological mechanisms [48]. Instead, colonies are selected for a simple ‘low-*Varroa*’ phenotype. In Seeley’s description, his Darwinian beekeeping is achieved by choosing a mite infestation threshold beyond which he deems a colony ‘doomed’ and culled or not used in further breeding regimes. Seeley presents this as an ‘acceleration’ of Darwinian natural selection, but we argue the two processes are significantly different. ‘Seeley-style’ breeding paradigms do not act in the same way as natural selection via survivor-stock as described by other honeybee researchers [50,51], and the evolutionary outcomes will not be the same. By selecting for low-mite parasitic load, Seeley’s culling approach does not permit the evolution of tolerance of *Varroa* in honeybees, only resistance. Currently, Seeley’s efforts in popularizing selection approaches for better bees [49] among beekeepers have been lost to a broader survivor-stock understanding of Darwinian beekeeping. Based on the cases of evolved tolerance discussed above, we predict this will have very different outcomes to Seeley’s specific mite-threshold-culling approach that will only select resistance.

The mechanistic underpinnings of how a Seeley-style breeding programme achieves mite resistance remain open to the whims of the evolutionary process. In this way, it remains a partial ‘black box’ in terms of mechanistic outcomes but not in terms of resistance outcomes. However, resistance can be bred by selecting specific behavioural-immunological phenotypes to control (resist) mites. Behavioural social-immunity mechanisms have been long-standing targets for selective breeding of honeybees in the face of *Varroa* [121,124]. For example, *Varroa*-sensitive-hygiene includes a suite of behaviours in which the honeybees can detect infested brood, uncap infected cells and remove parasitized brood [125,126]. Additionally, ‘mite-biter’ bees can bite and damage the mites to the point of decreasing the mite’s fitness [127]. By breeding for these specific behaviours, beekeepers can actively select resistance mechanisms that actively clear *Varroa* (see control, box 2).

We note that some associated behavioural mechanisms with current phenotypes of *Varroa*-resistant honeybees go against the current needs of commercial beekeepers. This includes smaller colony sizes, heightened defensiveness, an increased propensity to swarm, abscond or produce drones [106,128]. Mounting immune responses also have inherent physiological costs in the form of immunopathology [129]. There are elevated energetic requirements to engage in increased hygienic behaviours, aggression or swarming. Resistance is therefore costly to maintain evolutionarily without continued pathogen pressure as the costs of mounting effective immune responses trade off with life-history traits such as fecundity and development time [130]. A specific study using our described framework investigating the *scutellata-*hybrid (‘Africanized’ bees) escape in the Americas may prove fruitful [131].

Breeding bees that have characteristics of *Varroa* resistance, evolutionary longevity and viability in the agricultural system pose a significant challenge, which influences whether Seeley-style ‘mechanistic black box’ approaches are worthwhile compared with mechanism-first approaches. Advances in identifying genetic markers for some of these traits have been made [106,132] and used as part of selective breeding regimes. This underscores the achievability of aiming for truly resistant bees. Evidence suggests that there is significant repeatability in the mechanisms of *Varroa* resistance which can evolve [34,120], reinforcing the plausibility of achieving phenotypes that are almost entirely reflective of resistant, rather than tolerant, strategies. Additional mechanisms of mite resistance continue to be discovered. For example, the degree of immune response mounted by developing bees has been directly linked to *Varroa* fecundity, revealing additional physiological or immunological phenotypes that may control mite levels beyond just behavioural responses [5,133,134]. Understanding these diverse phenotypes provides multiple avenues for bee breeders to be purposeful, intentional and specific in their selection regime, and by that understanding and purpose, avoid the tragedy of tolerance.

## 5. Conclusion

The evolution of parasite tolerance in honeybees poses three tragedies: (i) tolerance represents an evolutionary end-state where more individuals may be dying from a parasite than was the case when the population was fully susceptible; (ii) increased spillover frequency in native bees owing to the greater number of infected honeybees in the landscape, each being more infectious owing to higher pathogen replication rates and (iii) when spillover occurs from honeybees to wild bees, the infection is more virulent in non-tolerant wild bees. Unfortunately, tolerance of *Varroa* and corresponding viruses is a potential outcome of current ‘survivor stock’ selection regimes, which we characterize as ‘tolerance-permissive’. Tolerance risks to both managed and wild bees can be circumvented by the selection explicitly for resistance to *Varroa* in managed honeybees. Resistance is the expected outcome of programmes selecting for any ‘mechanism-first’ bee breeding efforts and is also achievable through ‘mechanistic black box’ beekeeping which can be described as ‘Seeley-style Darwinian’ selection. While tolerance is dangerous, and, if left unaddressed, a very likely outcome in the honeybee system, it is not inevitable. Beekeeper education on why certain breeding programmes and approaches are more ecologically responsible may allow this critical livestock industry to both avoid the tragedies of tolerance for wild bees and mitigate current honeybee losses.

## Data Availability

This article has no additional data.
